# Supplementary figures and images for: The Isolation, Genetic Analysis and Biofilm Characteristics of Listeria spp. from the Marine Environment in China
Source: Microorganisms. 2023 Aug 27;11(9):2166. doi: 10.3390/microorganisms11092166 (PMC10535974; doi:10.3390/microorganisms11092166)

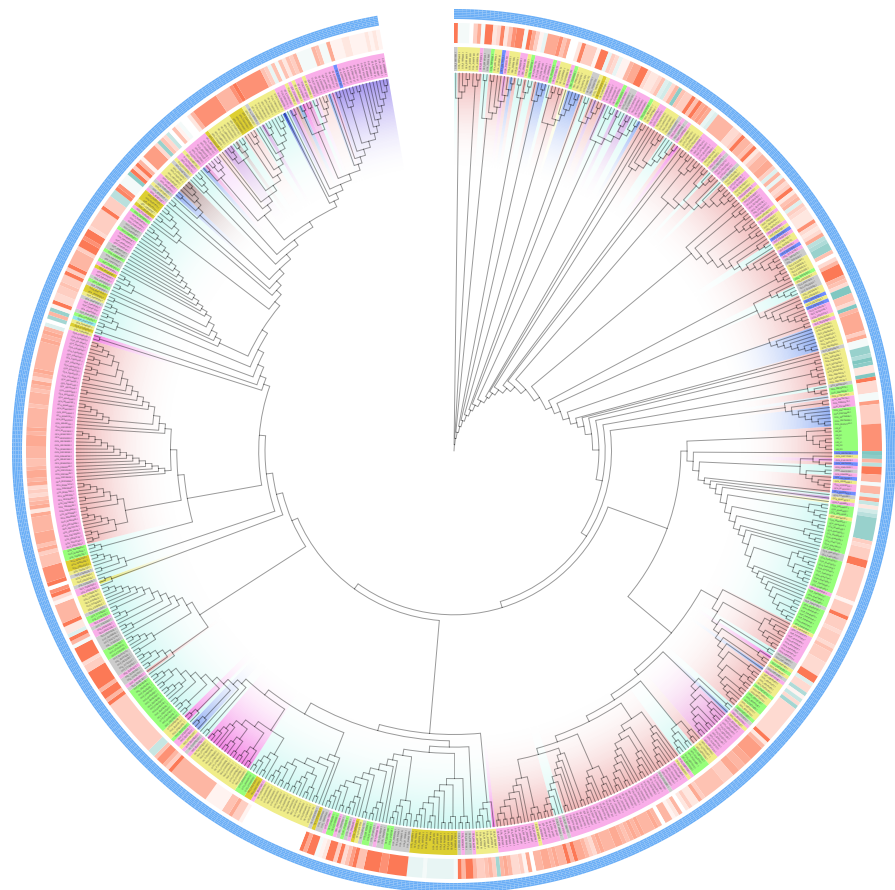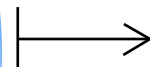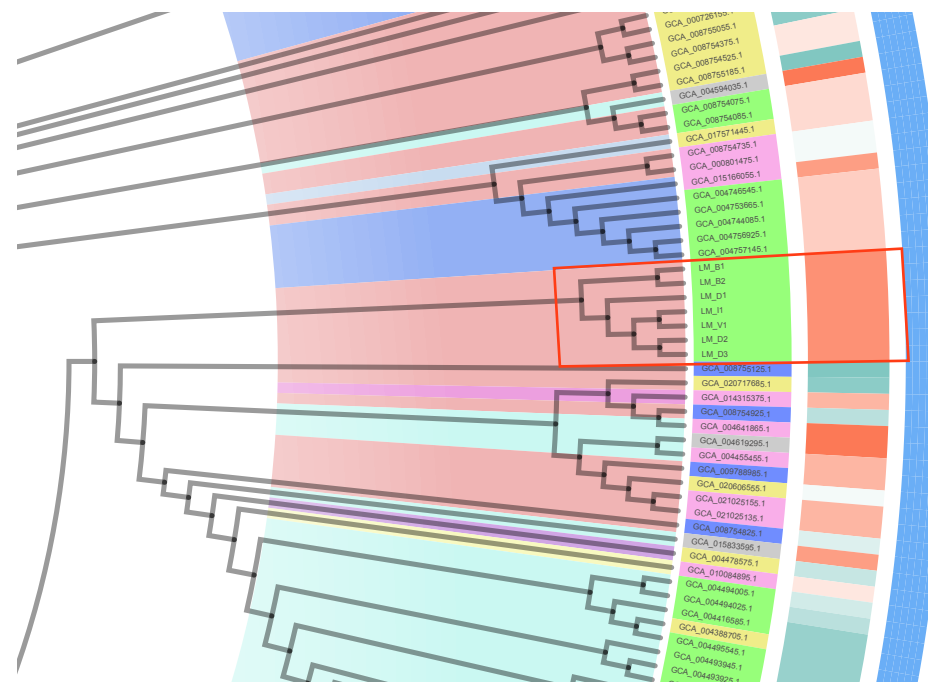

#### geographic location

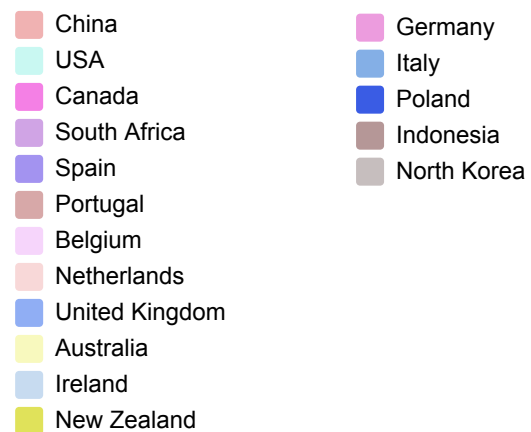

#### source

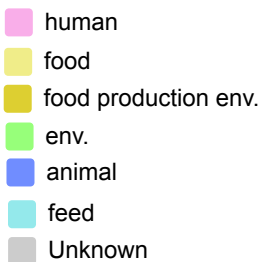

#### collection date

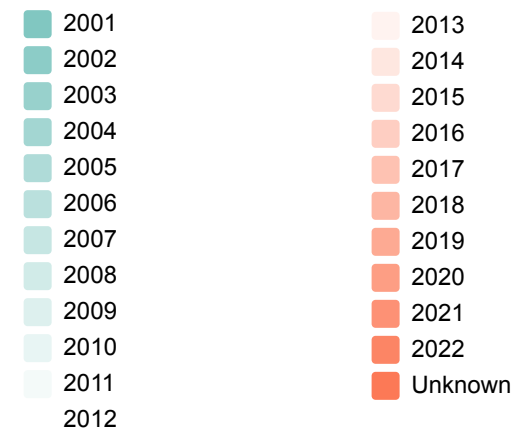

#### LIP-4

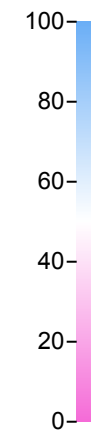

Supplement: Supplementary file 1 [file microorganisms-11-02166-s001.zip › Figure S1.pdf]

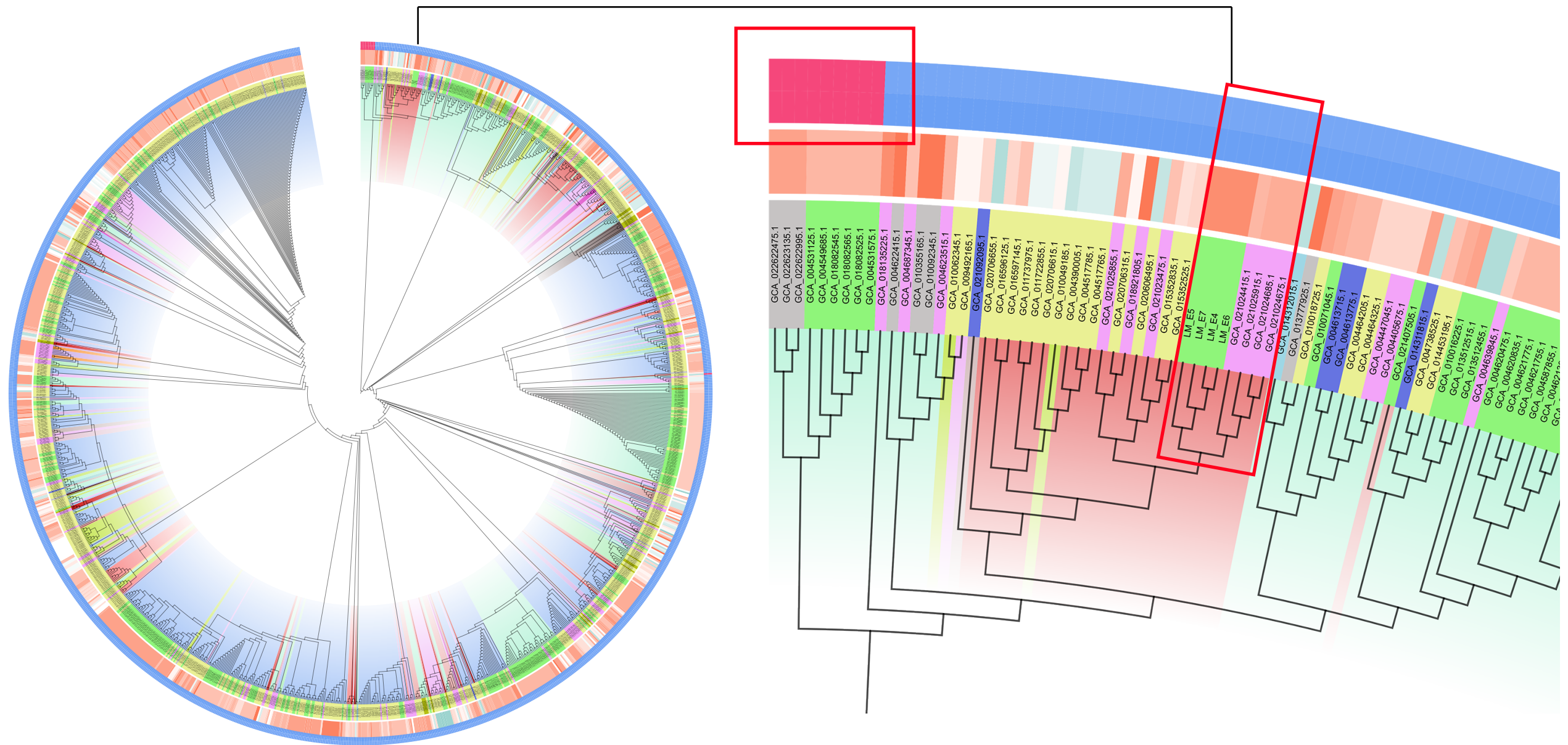

### geographic location

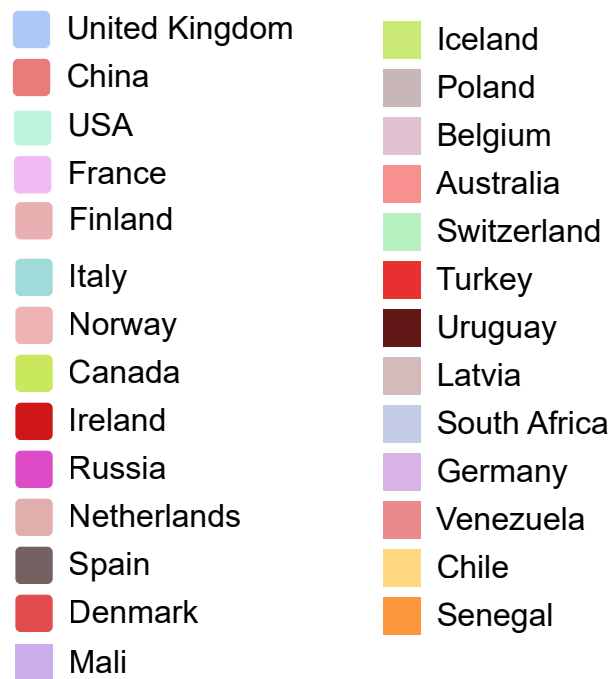

### source

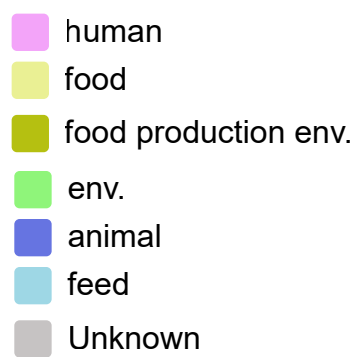

### collection date

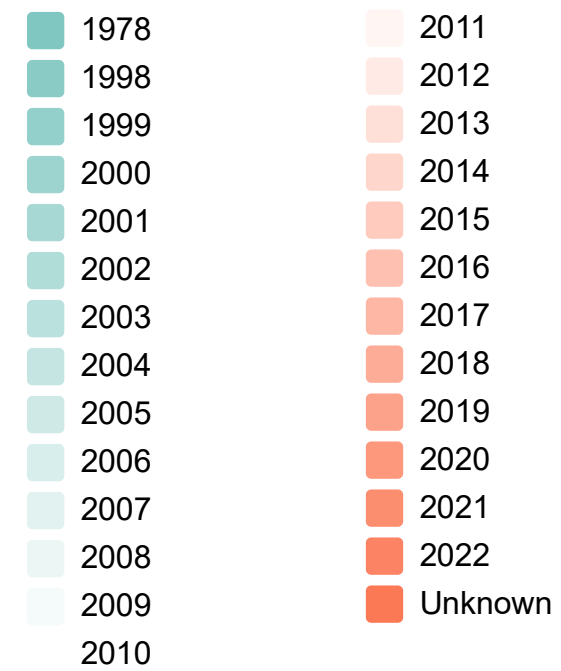

### SSI-2

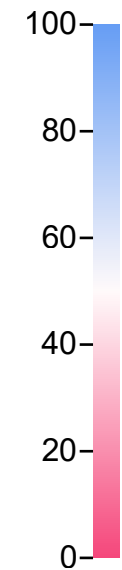

Supplement: Supplementary file 1 [file microorganisms-11-02166-s001.zip › Figure S2.pdf]

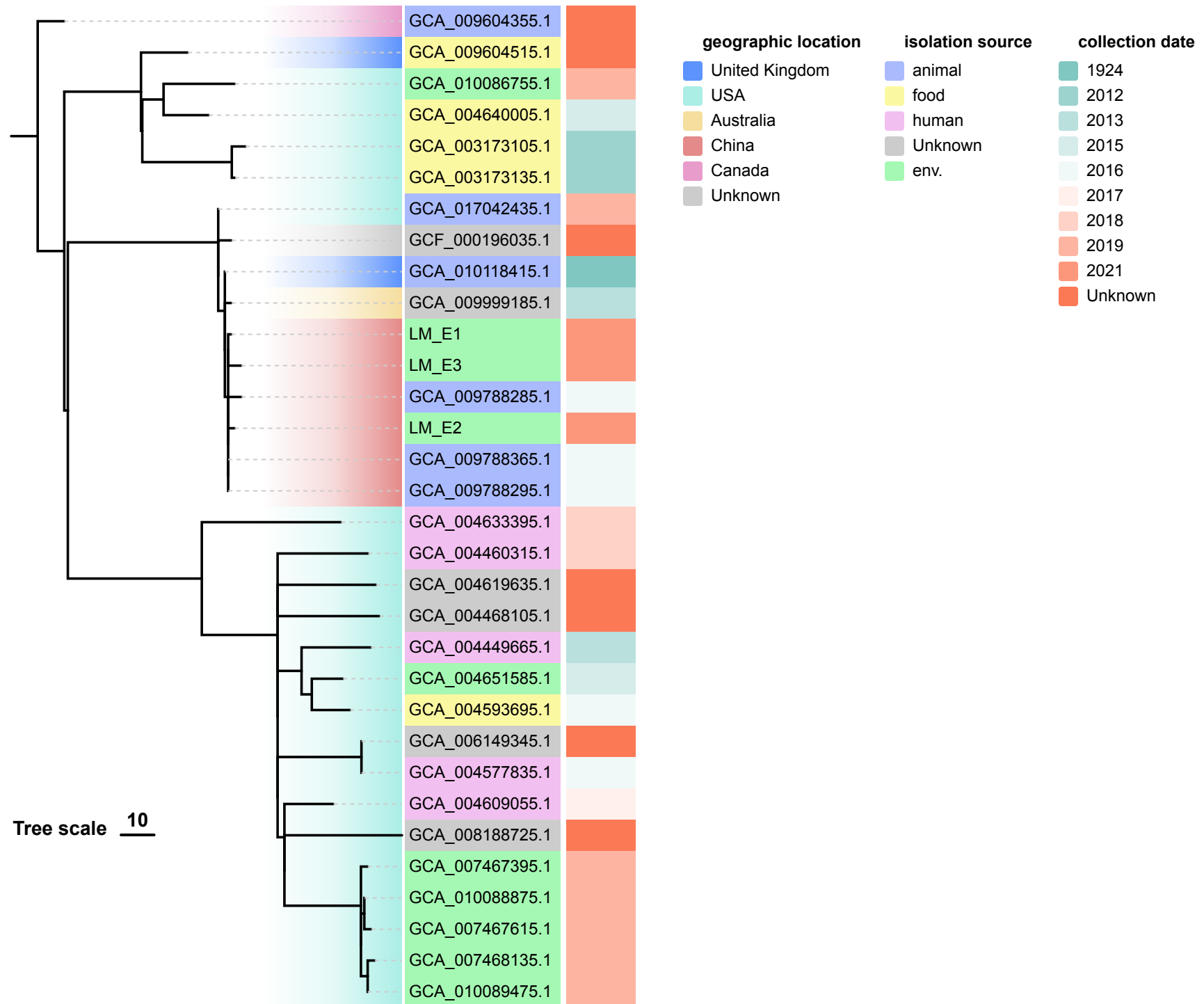

Supplement: Supplementary file 1 [file microorganisms-11-02166-s001.zip › Figure S3.pdf]

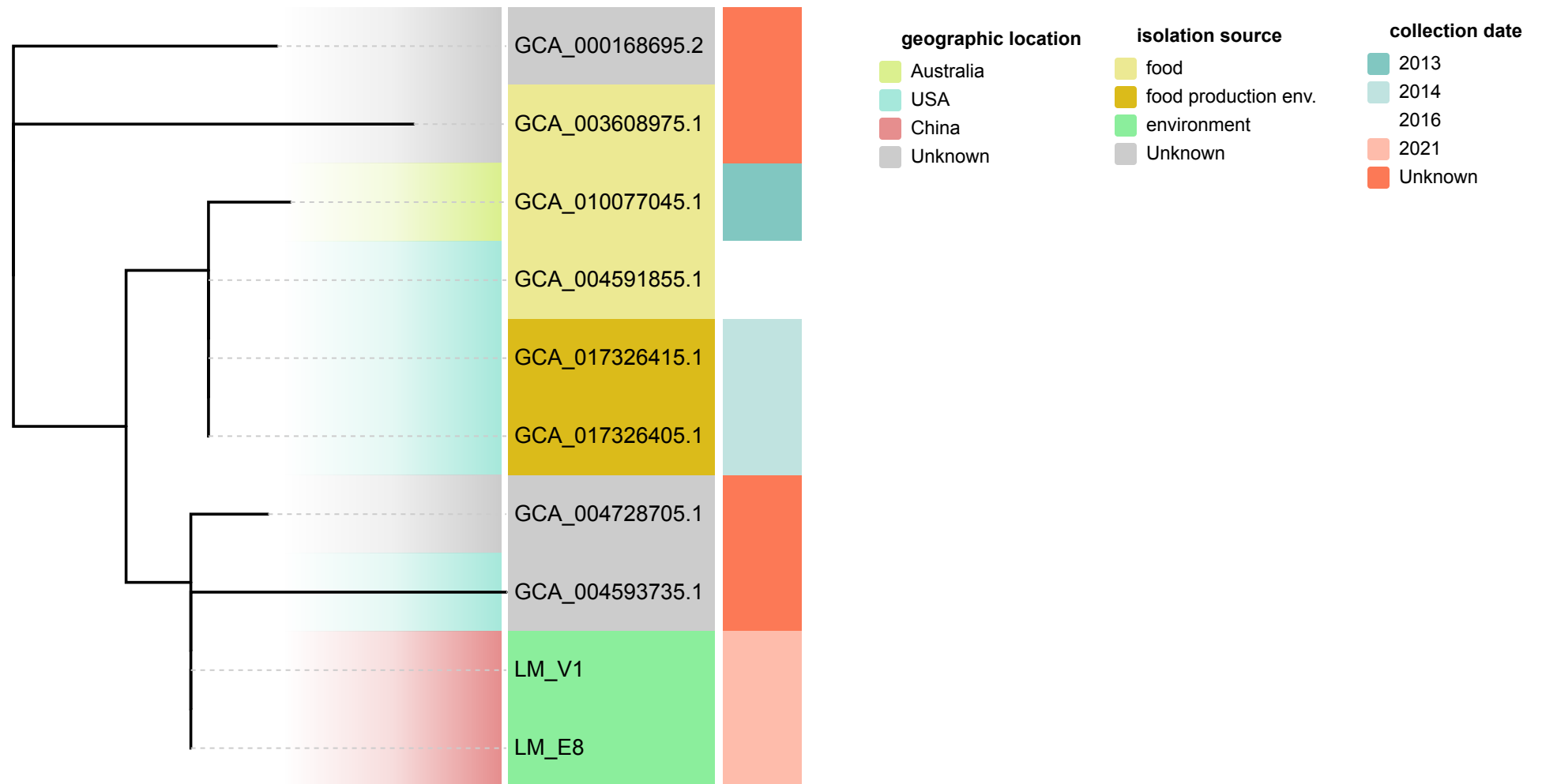

Tree scale 0.5

Supplement: Supplementary file 1 [file microorganisms-11-02166-s001.zip › Figure S4.pdf]
