# Supplementary material for: The Isolation, Genetic Analysis and Biofilm Characteristics of Listeria spp. from the Marine Environment in China
Source: Microorganisms. 2023 Aug 27;11(9):2166. doi: 10.3390/microorganisms11092166 (PMC10535974; doi:10.3390/microorganisms11092166)
Supplement: Supplementary file 1 [file Figure S5.pdf › Figure S5.pdf]

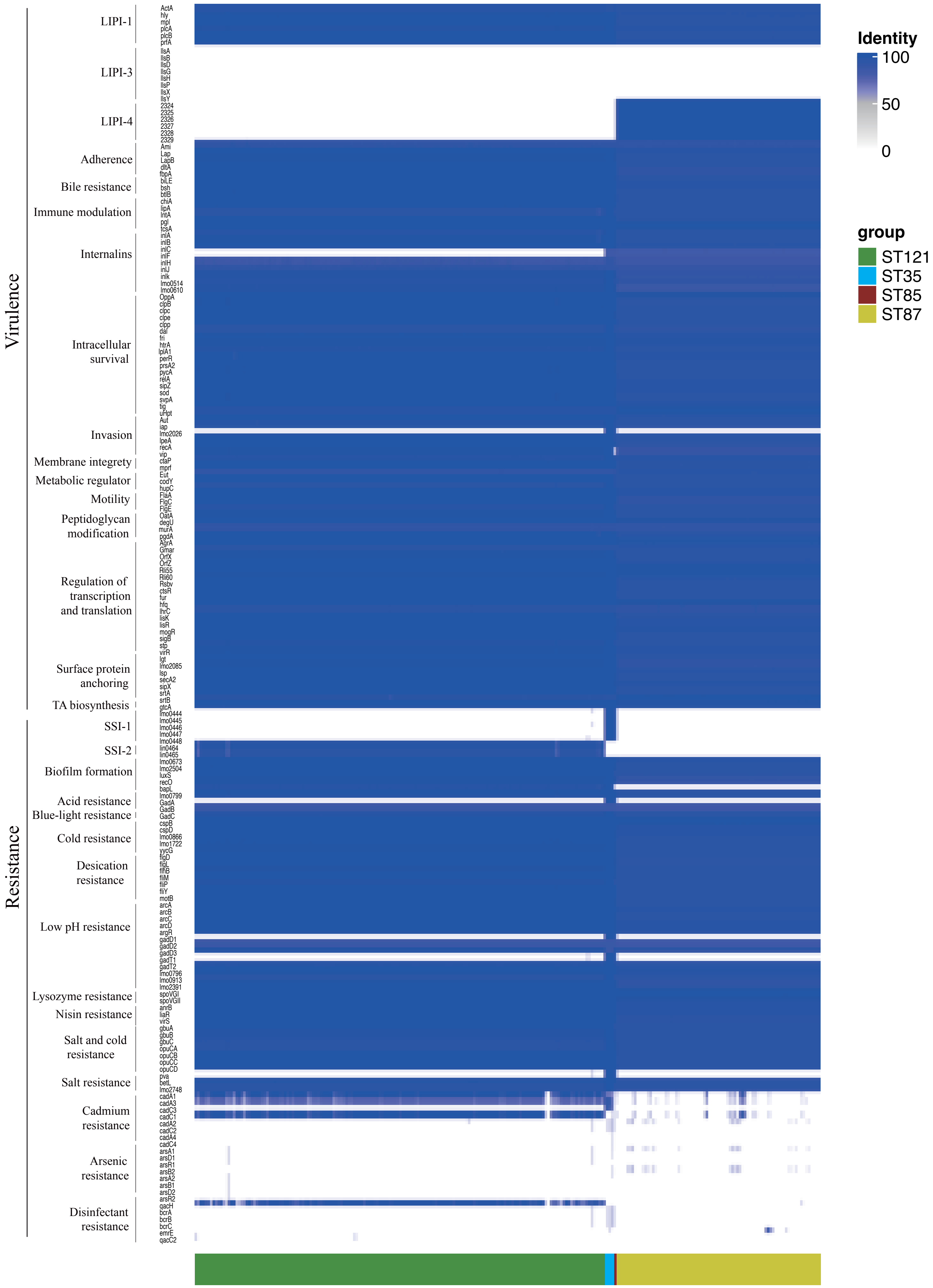
Virulence
LIPI-1
LIPI-3
LIPI-4
Adherence
Bile resistance
Immune modulation
Internalins
Intracellular survival
Invasion
Membrane integrety
Metabolic regulator
Motility
Peptidoglycan modification
Regulation of transcription and translation
Surface protein anchoring
TA biosynthesis
Resistance
SSI-1
SSI-2
Biofilm formation
Acid resistance
Blue-light resistance
Cold resistance
Desication resistance
Low pH resistance
Lysozyme resistance
Nisin resistance
Salt and cold resistance
Salt resistance
Cadmium resistance
Arsenic resistance
Disinfectant resistance
ActA
hly
mpl
plcA
plcB
prfA
llsA
llsB
llsD
llsG
llsH
llsP
llsX
llsY
2324
2325
2326
2327
2328
2329
Ami
Lap
LapB
dltA
fbpA
biLE
bsh
btlB
chiA
lipA
lntA
pgl
tcsA
inlA
inlB
inlC
inlF
inlH
inlJ
inlk
lmo0514
lmo0610
OppA
clpB
clpc
clpe
clpp
dal
fri
htrA
lplA1
perR
prsA2
pycA
relA
sipZ
sod
svpA
tig
uHpt
Aut
iap
lmo2026
lpeA
recA
vip
ctaP
mprf
Eut
codY
hupC
FlaA
FlgC
FlgE
OatA
degU
murA
pgdA
AgrA
Gmar
OrfX
OrfZ
Rli55
Rli60
Rsbv
ctsR
fur
hfq
lhrC
lisK
lisR
mogR
sigB
stp
virR
lgt
lmo2085
lsp
secA2
sipX
srtA
srtB
gtcA
lmo0444
lmo0445
lmo0446
lmo0447
lmo0448
lin0464
lin0465
lmo0673
lmo2504
luxS
recO
bapL
lmo0799
GadA
GadB
GadC
cspB
cspD
lmo0866
lmo1722
yycG
flgD
flgL
flhB
fliM
fliP
fliY
motB
arcA
arcB
arcC
arcD
argR
gadD1
gadD2
gadD3
gadT1
gadT2
lmo0796
lmo0913
lmo2391
spoVGI
spoVGII
anrB
liaR
virS
gbuA
gbuB
gbuC
opuCA
opuCB
opuCC
opuCD
pva
betL
lmo2748
cadA1
cadA3
cadC3
cadC1
cadA2
cadC2
cadA4
cadC4
arsA1
arsD1
arsR1
arsB2
arsA2
arsB1
arsD2
arsR2
qacH
bcrA
bcrB
bcrC
emrE
qacC2
Identity
100
50
0
group
ST121
ST35
ST85
ST87
